# Hysterosalpingographic, ultrasonographic and clinical profile of infertile women in Butembo, Eastern Democratic Republic of Congo

**DOI:** 10.11604/pamj.2023.46.105.39834

**Published:** 2023-12-14

**Authors:** Dieumerci Kaseso, Justine Valyananzighu, Olivier Mulisya, Baraka Munyanderu, Philemon Matumo, Charles Posite, Alexandre Amini, Ahuka Ona Longombe

**Affiliations:** 1 Department of Gynecology and Obstetrics, Faculty of Medicine, Catholic University of Graben, Butembo, Democratic Republic of Congo,; 2 Department of Gynecology and Obstetrics, Catholic University of Graben Teaching Hospital, Butembo, Democratic Republic of Congo,; 3 Department of Gynecology and Obstetrics, FEPSI Hospital, Butembo, Democratic Republic of Congo,; 4 Department of Gynecology and Obstetrics, Matanda Hospital, Butembo, Democratic Republic of Congo,; 5 Department of Urology, Muhimbili University of Health and Allied Sciences, Dar Es Salam, Tanzania,; 6 Department of General Surgery, University of Kisangani, Kisangani, Democratic Republic of Congo

**Keywords:** Ultrasonography, hysterosalpingography, infertility, women, DR Congo

## Abstract

**Introduction:**

infertility is a reproductive health issue in modern society. In developing countries, ultrasonography and hysterosalpingography (HSG) are first-line exams investigating infertility in women. It is a highly reported issue in Africa and is linked to abnormalities diagnosed by medical imagery investigations. Our research aimed to evaluate ultrasonography and HSG usage in female infertility investigation in eastern DR Congo, and to point out the most frequent lesions in infertile women in this area.

**Methods:**

it was a cross-sectional research. It included 1024 patients in four equipped hospitals with HSG and ultrasonography, who consulted from January 1^st^, 2019 up to December 31^st^, 2021. Data were collected from consultation dossiers and imagery protocols.

**Results:**

of 1024 patients, the mean age was 30.85±5.05 years, 41.79 % (n=428) had primary infertility and 57.71% (n=591) had secondary infertility with parity ranges 1.28±1.25, abortion 1.17±1.33. HSG usage rate was 26.85% (n=275) whereas ultrasonography was 66.01%(n=749). The prevalent diagnosed lesions were uterine myomas 10.51 % (n=71), polycystic ovary syndrome (PCOS) 8.28%(n=56), endometrial dysplasia 7.99% (n=54), ovarian cysts 5.03% (n=34) at ultrasonography and tubal obstructions 53.45 %(n=147), hydrosalpinx 4.73% (n=13), cervical impotence 3.27% (n=9), uterine synechias 2.55%(n=7), müllerian abnomalies 2.55%(n=7), uterine retroversion 2.18% (n=6) at HSG. History of upper genital infection was a risk factor aOR= 3.71, 95%CI 1,55-8,88; p <0.001 for tubal obstruction to HSG.

**Conclusion:**

regarding the high prevalence of tubal and uterine abnormalities in infertile women of eastern DR Congo, ultrasonography, and HSG should be more performed exams in clinical practice in low-income countries.

## Introduction

Infertility is defined as failure to get pregnant after 12 months or more of regular unprotected sexual intercourse [1,2]. Worldwide, 186 million people suffer from infertility, the majority in developing countries [3]. It affects 8 to 12% of reproductive-aged couples, with 20% to 35% due to women [4,5]. In women, infertility is caused by abnormalities of the ovaries, uterus, fallopian tubes, and the endocrine system [6]. After hormonal analysis, medical imagery is the main complementary exam in its investigation [7]. Hysterosalpingography (HSG) and ultrasonography serve as the main tools to explore the anatomy and function of the reproductive system and then to rule out causes and factors of infertility in women [8].

In the USA, 19% to 26% of women aged 15 to 49 years consult for infertility [9], with the most commonly diagnosed lesions: polycystic ovary syndrome (PCOS), endometriosis, and tubal anomalies respectively in 30%, 20% and 11% [10]. In France, around 150,000 women consult for infertility each year [11]. In Africa, infertility affects 21% of couples, with 37% due to women [12]; the most frequently reported causes are tubal obstructions 42% to 55%, uterine pathologies 10% to 15% and ovarian dystrophies 5 à 7% [13,14]. In Nigeria, 25% of women of reproductive age were reported infertile with tubal obstructions 39.5%, uterine abnormalities 30 % and ovarian cysts 13% in 2008 [15]; in Ghana, they reported 43.6 % tubal obstructions, 25.4 % pelvic adherence, 13.4% of hydrosalpinx, 5.4% of endometrial dysplasia in infertile women [16]; and in Cameroon tubal obstructions 36.6%, uterine abnormalities 12.5%, uterine myomas 6.7%, cervical impotence 5.8% and peritoneal adherence 5.8% [17]. In Democratic Republic of the Congo (DR Congo), they reported tubal obstructions 72.31%, peritoneal adherence 10.77%, uterine myomas 8.46%, uterine malformations 6.15% and uterine retroversion 4.62% in women with infertility in Kisangani [18]; and tubal obstructions and pelvic adherence 61.9%, ovarian dystrophies 41%, uterine pathologies (synechias, endometriosis and myomas) 4.8%, cervico-isthmic impotence 3% in Goma [19]. Reproductive system abnormalities and pathologies come as common problems in female infertility in African regions. Still, in some areas, few women undergo HSG and ultrasonography; many others use auto medication without any investigation [20]. Our research aimed to evaluate the rate of HSG and ultrasonography usage in the clinical investigation of infertility in women of Butembo DR Congo and the most frequently diagnosed lesions.

## Methods

**Study design and setting:** this research was conducted in Butembo, eastern DR Congo, in four referral hospitals: Teaching Hospital of Graben, Wanamahika Hospital, Matanda Hospital and FEPSI Hospital. The four hospitals selected have all a gynecologist and imaging equipment. The labels were « Zoncare-V3 2D» for ultrasonography in the four Hospitals and « Siemens Opti 150/12/50 C » for Rx HSG at Matanda Hospital and Wanamahika, and « Kimray Medical » for Rx HSG at Teaching Hospital of Graben and FEPSI.

**Study population:** our research was a descriptive cross-sectional that included 1024 patients aged 18 years to 45 years old who consulted for infertility in the 4 hospitals from January 1^st^ 2019 and December 31^st^, 2021. Included in our research, were all the patients aged from 18 to 45 years, living with a partner with regular sexual intercourse for 1 year or more, who consulted for infertility. Our sample was exhaustive, taking all the patients who fulfilled the inclusion criteria.

**Data collection:** data were collected from medical records of patients, with a complete protocol of HSG and/or ultrasonography.

**Statistical analysis:** they were processed with Epi Info version 7.2.4.0. We included 9 variables: age, marital status, origin, type of infertility, medical and surgical background, history of genital infections, diagnosed lesions at ultrasonography, and HSG. The khi-2 test and odds ratio served to evaluate dependence among variables.

**Ethical considerations:** participant's identity was kept secret, and our research didn´t use any invasive procedure. Data were collected after the approval of the Ethical Committee of each selected hospital.

## Results

**General characteristics of the study population:** our research included 1024 patients. The use of ultrasonography was at 66.01% (n=676) and HSG was at 26.85% (n=275). The age of patients ranged from 18 to 45 years with an average of 30.85 years; 97.46% were married and 2.54 % were single; 89.26% were from urban areas and 10.74 % from rural areas, 40.33% (n=413) consulted for primary infertility and 59.67% (n=611) had secondary infertility. The demographic characteristics and the histories of patients are presented in Table 1, depending on the type of infertility.

**Table 1:** demographic characteristics and patients’ histories based on type of infertility

Parameter	Frequency	Primary infertility	Secondary infertility
		n= 413	n= 611
Age*	-	29.47±5.35	31.84±5.05
Parity*	-	0(0%)	1.28±1.25
History of abortion*	-	0(0%)	1.17±1.33
Tubal surgery (TEP)	67(6.54%)	17 (25.37%)	50 (74.62%)
Post-abortum curettage	117(11.42%)	0 (0%)	117 (100%)
Cystectomy	82(8.00%)	46 (56.09%)	36 (43.90%)
Fibroid myomectomy	37(3.61%)	20 (10.15%)	177 (89.84%)
Others	90(8.78%)	34 (37.77%)	56 (62.22%)

* Mean ± standard deviation TPE: tubal ectopic pregnancy

**The results of ultrasonography:** the results of ultrasonography are presented in Table 2. Out of 676 cases of ultrasonography, 33.58% (n=227) were abnormal and 66.42% (n=449) were normal. The prevalent diagnosed lesions were uterine myomas 10.51 % (n=71), polycystic ovary syndrome (PCOS) 8.28% (n=56), endometrial dysplasia 7.99% (n=54), ovarian cysts 5.03% (n=34). Uterine hypoplasia was observed in 1.77% (n=12) patients. Uterine anomalies were most prevalent at 20.27% (n=137).

**Table 2:** abnormalities observed on ultrasonography based on type of infertility

Ultrasonographic results		Frequency (N= 676)	Primary infertility (n =285)	Secondary infertility (n =391)	p-value
Normal		449 (66.42)	191 (42.54)	258 (57.46)	0.77
Uterine Lesions		137 (20.27)	56 (40.87)	81(59.13)	0.73
	Myomas	71 (10.51)	34 (47.89)	37 (52.11)	0.31
	Endometrial dysplasia	54 (7.99)	13 (24.07)	41 (75.93)	0.01
	Uterine hypoplasia	12 (1.77)	9 (75.00)	3 (25.00)	0.02
Ovarian lesions		90 (13.31)	38(42.22)	52(57.78)	0.98
	Ovarian cysts	34 (5.03)	13 (38.24)	21 (61.76)	0.63
	PCOS*	56 (8.28)	25 (44.64)	31 (55.36)	0.69

* POCS: polycystic ovary syndrom

**The results of HSG:** the results of HSG are presented in Table 3. Of 275 cases of HSG, 195 (70.91%) were abnormal and 80 (29.09%) were normal. The abnormalities observed were tubal obstructions 53.45 %(n=147), hydrosalpinx 4.73% (n=13), cervical impotence 3.27% (n=9), uterine synechias 2.55%(n=7), müllerian anomalies 2.55%(n=7), uterine retroversion 2.18% (n=6) at HSG. The most prevalent lesions were tubal obstructions, 53.45% (n=147).

**Table 3:** abnormalities observed at HSG based on type of infertility

Hysterosalpingography (HSG) results			Primary infertility	Secondary infertility	p-value
		N=275	n =128	n=147	
Normal		80 (29.09)	38 (47.50)	42 (52.50)	0.83
Tubal lesions		160 (58.18)	77 (48.12)	83 (51.88)	0.53
	Tubal obstruction	147 (53.45)	70 (47.62)	77 (52. 38)	0.7
	Hydrosalpinx	13 (4.73)	7 (53.85)	6 (46.15)	0.58
Uterine abnomalies		35 (12.73)	13 (37.14)	22 (62.86)	0.23
	Myomas	6 (2.18)	4 (66.67)	2 (33.33)	0.31
	Uterine synechias	7 (2.55)	1 (14.29)	6 (85.71)	0.08
	Müllerian anomalies	7 (2.55)	5 (71.43)	2 (28.57)	0.18
	Cervical impotence	9 (3.27)	2 (22.22)	7 (77.78)	0.13
	Uterine retroversion	6 (2.18)	1 (16.67)	5 (83.33)	0.13

**Factors associated with tubal obstruction:** the specific risk for tubal obstructions at HSG based on the history of genital infections is shown in Table 4. There was a significant risk of 3.71 (aOR= 3.71, 95%CI 1,55-8,88; p <0.001) between the history of upper genital infection and the presence of tubal obstruction at HSG in patients.

**Table 4:** risk of tubal obstructions at HSG based on history of genital tract infections

History of genital infections		Total	Tubal obstructions	%	OR	IC à 95 %	
		N=275	n=147	53.45		Lim <	Lim >
None		103	48	46.6	0.64	0.39	1.05
Genital infection		172	99	57.56	1.55	0.95	2.53
	Upper	33	26	78.79	3.71	1.55	8.88
	Mixed	100	55	55	1.1	0.67	1.8
	Low	39	18	46.15	0.71	0.36	1.4

Hysterosalpingography (HSG)

## Discussion

Our research aimed to evaluate the rate of HSG and ultrasonography usage in the clinical investigation of infertility in women of Butembo DR Congo and the most frequently diagnosed lesions. It included 1024 patients who consulted for infertility; the average age was 30.85 years. Ultrasonography usage was 66.01% and HSG 26.85%. The most prevalent findings at ultrasonography were myomas at 10.50%, endometrial dysplasia at 7.99%, uterine hypoplasia at 1.78% and tubal obstructions at 53.45%, hydrosalpinx 4.73%, cervical impotence at 3.27 % at HSG. History of upper genital infection was a risk factor aOR= 3.71, 95%CI 1,55-8,88; p <0.001) for tubal obstruction to HSG. In general, female fertility decreases from the age of 35 years, due to ovulatory dysfunctions and other hormonal pathologies [21]. Our results were similar to other research in Kisangani DR Congo, Uganda, Cameroon, and Ghana with average ages between 31.7 to 33.52 years [16-18,22]. The rate of ultrasonography usage was 66.01% in our research and 26.85% for HSG. In normal conditions, these exams should be performed as the first line in female infertility investigation after hormonal screening [23]. Nevertheless, the low rate of HSG use in clinical practice may be related to the economic burden of this exam and its invasive character in our context; by the way, Maggie Williams *et al*. [24] in the United Kingdom and La Fianza A *et al*. [25] in Italy reported that HSG constitutes a source of anxiety for patients due to its invasive aspect, besides it is an economic burden for African health system [26]. However, there was no major difference in proportion neither between a number of ultrasonographies nor HSG performed, based on the sociodemographic background of patients.

Clinical history (Table 1), came to be a factor in infertility onset in women. It is the case of endo-uterine curettage, tubal surgery, or genital infection [27], especially endo-uterine curettage is reported to be a risk factor of Asherman syndrome, characterized by endo-uterine adherence after traumatic or inflammatory reactions [28,29]. Regarding the prevalence of reproductive system anomalies on ultrasound Table 2), uterine anomalies were most prevalent (20.27%), with a significant association between endometrial dysplasia and secondary infertility (p=0.01) and between uterine hypoplasia and primary infertility in patients (p=0.02). The specific uterine lesions on ultrasound were 20.27% (myomas 10.50%, endometrial dysplasia 7.99%, uterine hypoplasy 1.78%), ovarian lesions 13.3% (PCOS 8.28%, and ovarian organic cysts 5.03%) and a normal ultrasonographic result 66.42%. As it has been found in our research, Porcu G and Heckenroth H reported a prevalence of 5 to 10% of myomas in infertile females [30]. It is nevertheless an exceptionally isolated cause of infertility [31]. Myomas can lead to infertility by hampering the normal process of nidation and abortion induction by reducing the uterine capacity to carry the pregnancy [32]. Otherwise, among ovarian lesions, PCOS was most prevalent at 8.28%. This was similar to the results of Brassard *et al*. where PCOS represented 10 % of causes of infertility in women, in particular primary infertility [33]. Polycystic ovary syndrome (PCOS) leads to infertility by anovulation; thus 50% of women with PCOS present primary infertility and 25% secondary infertility; it is reported the cause of 70% of infertility by ovulation [34,35]. In the literature, it is reported a prevalence of 5% of adenomyosis in female infertility [36]. Yet, our research didn´t report any. This can be related to ultrasonographic sensitivity in our context, its diagnosis requires supplementary skills of the radiologists.

For HSG results, we found the following results (Table 3): tubal obstructions 53.45%, hydrosalpinx 4.73%, cervical impotence 3.27%, endometrial polyps 2.8%, uterine synechia 2.55% and uterine abnormalities 2.55%. These results were similar to those reported in Kisangani, DR Congo, where a prevalence of tubal obstructions of 72.31% was reported at HSG in infertile women [18]; and in Goma Mushabaa K et Labama L reported a prevalence of 67.6% of tubal obstructions in etiology of female infertility [19]. In other African countries: in Ghana, Nigeria, Cameroon, and Uganda tubal obstructions were reported respectively at a rate of 43.50%, 30.01%, 36.60%, and 52.90 % in infertile females [12,16,17,22]. Hysterosalpingography (HSG) comes as an important exam taking into account the high proportion of tubal factors in infertile females throughout African countries [16,17,37]. Many factors explain this high prevalence, in particular concern, genital infection (Table 4) constituted a risk factor of tubal obstruction with 3.71 à IC 95% (1.55-8.88) risk of tubal occlusion in patients with a history of upper genital infection. In general, it is assumed that genital infections represent a high risk of post-infectious adherence, particularly the infection of chlamydia trachomatis [38,39]. That way, our results fitted with Egbe TO *et al*. who reported a risk of 7.3 of tubal occlusion in patients with a history of genital infections in Cameroon, mostly due to complications of postpartum or post-abortum in low asepsis settings [40]. In this research, we had the limitation of low rate of HSG performed compared to ultrasonography, to get more statistical significance. Nevertheless, it gave an overview on the clinical investigation of infertility in Eastern DR Congo and the prevalence of subsequent lesions in infertile women. This can serve as a springboard for prospective studies and an orientation for medical professionals in this area, investigating infertility.

## Conclusion

Our research included 1024 patients who consulted for female infertility in four gynecological referral hospitals of Butembo, eastern DR Congo, from the 1^st^ January 2019 to 31^st^ December 2021. The rate of ultrasonography use was higher than HSG use. The most prevalent lesions in our patients were uterine myomas and PCOS at ultrasonography; tubal obstructions and hydrosalpinx at HSG. Tubal obstructions came as a prevalent lesion in infertile women in Butembo, with genital infection as a major risk.

### What is known about this topic

- *Infertility in women is an emerging issue in Africa;*
- *Hysterosalpingography (HSG) is less performed in investigation of infertility in some African areas;*
- *Infertility is reported to be linked in Africa highly to tubal abnormalities, investigated by HSG*.

### What this study adds

- *Both ultrasonography and hysterosalpingography (HSG) are important exams to conduct in women´s infertility investigation in Africa, to explore the uterus, ovaries and fallopian tube, respectively;*
- *Hysterosalpingography (HSG) is less performed than ultrasonography in daily clinical investigation of infertility in women, in some African areas;*
- *Polycystic ovary syndrome (PCOS), uterine myomas, and tubal obstructions are frequent lesions in infertile women in some African areas*.
